# Methamphetamine Ingestion Misdiagnosed as *Centruroides sculpturatus* Envenomation

**DOI:** 10.1155/2015/320574

**Published:** 2015-01-14

**Authors:** Joshua Strommen, Farshad Shirazi

**Affiliations:** ^1^Department of Emergency Medicine, Carl R Darnall Army Medical Center, 36000 Darnall Loop, Fort Hood, TX 76554, USA; ^2^Arizona Poison & Drug Information Center (APDIC), University of Arizona College of Pharmacy, Tucson, AZ 85721, USA; ^3^Arizona Emergency Medicine Research Center, University of Arizona College of Medicine, Tucson, AZ 85721, USA

## Abstract

The authors present a case report of a 17-month-old female child who ingested a large amount of methamphetamine that looked very similar clinically to a scorpion envenomation specific to the southwestern United States by the species *Centruroides sculpturatus*. The child was initially treated with 3 vials of antivenom specific for that scorpion species and showed a transient, though clinically relevant neurologic improvement. Her clinical course of sympathomimetic toxicity resumed and she was treated with intravenous fluids and benzodiazepines after blood analysis showed significant levels of d-methamphetamine. This case report is to specifically underline the clinical confusion in discerning between these two conditions and the realization of limited and/or expensive resources that may be used in the process.

## 1. Introduction

Pediatric evaluation and treatment of* Centruroides sculpturatus* envenomation is well documented in recent literature. Using Centruroides (Scorpion) Immune F(ab′)2 Equine Injection as a treatment for* C. sculpturatus* envenomation was shown to be consistently effective, with only minimal side effects that include vomiting, fever, and rash as the most common manifestations. The Centruroides (Scorpion) Immune F(ab′)2 Equine Injection, also known as Anascorp, was used to treat over 1500 adult and pediatric patients alike, for known* C. sculpturatus* envenomation. Greater than 95% of these patients had symptom resolution within four hours after initiating Anascorp compared to a 3.1% resolution in a historical control database from 1990 to 2003 where only supportive care was given. Out of the 1500 patients mentioned above, none were diagnosed with the full serum sickness syndrome after receiving Anascorp [1]. Envenomation of pediatric patients by this specific type of scorpion, which has only been documented in the southwestern United States (US) and northern Mexico, is not uncommon. It is diagnosed by very specific characteristics, which, if not careful, can be mistaken for methamphetamine toxicity.

Unluckily, there is a predominance of methamphetamines in the same geographic area of the US, as the endemic locale of the* C. sculpturatus*. Illicit methamphetamine production and distribution in the United States has its historical origin from producers along the borders of both Mexico and California [2]. According to the United States Drug Enforcement Agency, for the calendar year 2012, just less than 11,000 kilograms of methamphetamine was seized along the Southwestern United States border with Mexico. This is the highest amount ever recorded. Arrestee data show stable rates of testing positive for methamphetamines in the western and southwestern United States versus the rest of the country, which reveals its geographic predominance and areas with higher rates of use [3].

Only two previously published case reports demonstrate cases of misdiagnosis of* C. sculpturatus* envenomation with what was actually a methamphetamine ingestion [4, 5]. However, none of the prior case reports of methamphetamine ingestion involve a patient who received the recommended full dose of antivenom and then showed transient neurologic improvement. The authors report a case of a 17-month-old female who had clinical improvement in neuromuscular hyperactivity and cranial nerve involvement after three vials of Anascorp for a suspected scorpion envenomation when, in fact, the patient had a methamphetamine intoxication. This observation of clinical improvement may further complicate the process of diagnosing the correct condition, in addition to the existing diagnostic dilemma of discerning methamphetamine toxicity versus a* C. sculpturatus* envenomation in nonverbal pediatric patients.

## 2. Case

A 17-month-old female with no previous medical problems presented to a community Emergency Department (ED) in Tucson, AZ, because of acute onset irritability, twitching throughout her entire body, and diaphoresis. On arrival to the ED her triage vital signs were documented as a heart rate of 122, a respiratory rate of 24, oxygen saturation of 90%, and rectal temperature of 99.7°F. Physical examination done in the ED revealed an alert and oriented female child with agitation and tremors. Her Glasgow Coma Scale was 15 and she had no derangement in her blood glucose. Pupils were equally reactive with 5-6 mm of mydriasis along with rotary nystagmus. Extraocular movements were intact. The patient's oral exam was consistent with excessive salivation, although there were no pooling secretions in the pharynx. The patient was in sinus tachycardia with no obvious murmurs and had clear breath sounds bilaterally with tachypnea. There were no obvious lesions, bruises, bites, abrasions, or erythema noted on skin exam.

The patient had presenting symptoms of abnormal eye movements, excessive salivation, and tachypnea, all of which are direct indications per the package insert for Anascorp to initiate administering the antivenom [1]. Along with the concomitant geographic setting of an Emergency Department in the Southwest US,* C. sculpturatus* envenomation was suspected as a possible diagnosis. Upon questioning, the patient's mother stated that she had seen scorpions multiple times within their home. Based on the concern for envenomation, the recommended three vials of Anascorp were given to the patient after consulting with the local Poison Center within minutes of the initial evaluation. Since scorpion envenomation was the presumed diagnosis and Anascorp was given, benzodiazepines were withheld for her agitation at this time. Both subjective and objective improvement was noted in the clinical condition of the patient within 30–40 minutes. Her nystagmus and secretions resolved, though she continued having generalized tremors.

However, the patient remained tachycardic and developed a rectal temperature of 102.0°F. Upon further questioning of the patient's mother, she revealed a history of methamphetamines being present and used at the caretaker's home in the past. While the route of ingestion for the child was unknown, the ingestion itself was confirmed by a positive urinalysis for methamphetamines. An expansive set of toxicology labs and routine chemistries were obtained and evaluated, in addition to the urine drug screen, all of which showed no clinically pertinent abnormalities. The patient was then transferred to a tertiary care facility due to her continued symptoms of fever, tachycardia, agitation, and tremors.

At the tertiary care center, the patient remained with a sympathomimetic toxidrome. Evaluation by the toxicology team showed no clonus, rigidity, fasciculation, excessive salivation, or nystagmus. Per the parents, the patient never exhibited any behavior consistent with severe, localizing, extremity, or truncal pain typical for scorpion envenomation. Nor did the exam of the child on arrival show any direct or indirect skin findings consistent with a scorpion sting. The patient remained tachycardic, hypertensive, febrile, irritable, with mydriasis, and diaphoresis intermittently for 48 hours after admission. A noncontrast brain CT was completed to rule out the presence of spontaneous bleeding, which can be associated with severe methamphetamine ingestion. She had no clinically apparent seizure activity and had a negative EEG. The only pertinent lab abnormality was a creatine kinase of 1467 IU/L, but the patient never developed acute kidney injury during her hospital course. The patient was treated symptomatically with benzodiazepines and intravenous fluids until she returned to baseline. She was discharged on hospital day 8. Her extended stay was contributed to her workup, her treatment with several days worth of benzodiazepines, and social work services. Several weeks later lab results from the initial Emergency Department evaluation revealed blood levels consistent with d-methamphetamine greater than 61,000 ng/mL and amphetamine levels greater than 21,000 ng/mL. The patient was never readmitted with any further sequelae from this ingestion per our documentation and records.

## 3. Discussion

There have been four reported cases documented in two separate case reports in peer-reviewed literature of methamphetamine ingestion by a toddler, appearing clinically similar to a scorpion envenomation and initially being treated as such [4, 5].* C. sculpturatus* venom contains a number of substances including neurotoxin, acetylcholinesterase, serotonin, histamine, protease inhibitors, phospholipases, hyaluronidase, and mucopolysacharides [6]. When present at toxic levels, the neurotoxin causes sodium channel inactivation, allowing an open state of the sodium channel, causing a prolonged action potential at the axonal membrane. This repeated depolarization results in an abundance of acetylcholine in the neuromuscular junction in both the central and peripheral nervous system. Cranial nerve abnormalities, bulbar dysfunction, neuromuscular hyperactivity, and dysautonomias are sensitive criteria for diagnosing this condition. Very specific findings reported by Suchard and Curry include tongue fasciculation, hypersalivation, slow and conjugate roving eye movements, and purposeless, uncoordinated motor agitation [7].

Methamphetamines have a distinct characteristic of enhancing both the central and peripheral nervous system excitatory pathways. There are two stereoisomers of methamphetamine, D-methamphetamine and L-methamphetamine. The L isomer is mostly a peripherally acting substance with alpha-adrenergic activity. It has been used in a number of products that serve as decongestants. The D isomer is the substance that has a central nervous system stimulant effect over 3–5 times more potent than that of its L counterpart. In addition when comparing the half-life of D-methamphetamine to another substance of abuse, cocaine, D-methamphetamine has a half-life of 10–12 hours versus two hours for cocaine [2]. The excitatory pathways are initiated when centrally located presynaptic monoamine reuptake transporters bind methamphetamine and release substances, which include dopamine, norepinephrine, and serotonin into the synapse.

Movement disorders from increased methamphetamine use or excessive nervous system stimulation can be associated with repetitive, stereotyped choreoathetoid movements, seizures, and tremors. Catecholamines are also significantly elevated from the adrenal medulla and postganglionic sympathetic nerve stimulation, which are responsible for causing tachycardia, mydriasis, and hypertension. The abnormal behavioral components of intoxication include aggression, psychosis, hypersexuality, and hallucinations [8]. They all stem from the dopaminergic and serotonergic alterations of the central nervous system.

Astute clinical observation and history of present illness, along with blood and/or urine toxicology screen, have revealed the true identity of the inciting agent in all reported cases. In this case, the patient had decreased cranial nerve dysfunction within 40 minutes after giving the Anascorp. The neuromuscular dysfunction of shaking, jerking, and tremors of the extremities remained however. It is plausible that this finding is due to the Anascorp administration, as a typical response time for antivenom is 30–60 minutes. The mechanism of action of Anascorp is by equine derived, venom-specific F(ab′)2 fragments of immunoglobulin G, which bind to specific toxins within bark scorpion venom. Usually four hours after administration of the antivenom, all symptoms should be resolved if the true etiology is bark scorpion envenomation [1].

In all previous case reports, only one vial of antivenom was given which yielded no clinical response, hence the mandatory search for an alternate diagnosis, whereas in this case the patient received the entire three recommended vials. Understandably, the etiology is not scorpion venom, but methamphetamine. Though as previously stated, the patient had reversal of some of her symptoms. Most notable was the disappearance of her rotary nystagmus and her oral secretions with the antivenom at a dose of three full vials. Additionally, there were also no adverse signs or symptoms to include rash, vomiting, or anaphylaxis after receiving the Anascorp.

The authors postulate that there was some amount of protein binding by the F(ab′)2 present in the blood, with the methamphetamine, causing a decrease in symptoms and clinical severity of the existing catecholamine surge. The authors realize certain questionable aspects of this theory. The most obvious incongruences are that the F(ab′)2 antibody is supposed to bind a protein which is magnitudes of size larger than the size of a methamphetamine molecule. There is also a huge discrepancy in cost between Anascorp and benzodiazepines. So to suggest that if this pharmacological interaction does exist between Anascorp and methamphetamine, we actually suggest its use would not be economically sensible. Currently there is an ongoing study in mice looking into this interaction with Anascorp and methamphetamine toxicity by one of the coauthors. The underlying point of this case and its findings are to highlight for the emergency medicine physician and pediatrician the ease with which these two clinical scenarios may be confused and some specific findings that are useful in detecting which problem actually exists.
